# Quetiapine has an additive effect to triiodothyronine in inducing differentiation of oligodendrocyte precursor cells through induction of cholesterol biosynthesis

**DOI:** 10.1371/journal.pone.0221747

**Published:** 2019-09-06

**Authors:** Jaime Gonzalez Cardona, Matthew D. Smith, Jingya Wang, Leslie Kirby, Jason T. Schott, Todd Davidson, Jodi L. Karnell, Katharine A. Whartenby, Peter A. Calabresi

**Affiliations:** 1 Department of Neurology, Johns Hopkins School of Medicine, Baltimore, Maryland, United States of America; 2 Early Respiratory, Inflammation and Autoimmunity, R&D Biopharmaceuticals, AstraZeneca, Gaithersburg, Maryland, United States of America; 3 Viela Bio, Gaithersburg, Maryland, United States of America; 4 Department of Neuroscience, Johns Hopkins School of Medicine, Baltimore, Maryland, United States of America; Instituto Cajal-CSIC, SPAIN

## Abstract

Multiple sclerosis (MS) is characterized by demyelinated lesions in the central nervous system. Destruction of myelin and secondary damage to axons and neurons leads to significant disability, particularly in people with progressive MS. Accumulating evidence suggests that the potential for myelin repair exists in MS, although for unclear reasons this process fails. The cells responsible for producing myelin, the oligodendrocytes, and their progenitors, oligodendrocyte precursor cells (OPCs), have been identified at the site of lesions, even in adults. Their presence suggests the possibility that endogenous remyelination without transplantation of donor stem cells may be a mechanism for myelin repair in MS. Strategies to develop novel therapies have focused on induction of signaling pathways that stimulate OPCs to mature into myelin-producing oligodendrocytes that could then possibly remyelinate lesions. We have been investigating pharmacological approaches to enhance OPC differentiation, and have identified that the combination of two agents, triiodothyronine (T3) and quetiapine, leads to an additive effect on OPC differentiation and consequent myelin production via both overlapping and distinct signaling pathways. While the ultimate production of myelin requires cholesterol biosynthesis, we identified that quetiapine enhances gene expression in this pathway more potently than T3. Two blockers of cholesterol production, betulin and simvastatin, reduced OPC differentiation into myelin producing oligodendrocytes. Elucidating the nature of agents that lead to complementary and additive effects on oligodendrocyte differentiation and myelin production may pave the way for more efficient induction of remyelination in people with MS.

## Introduction

The etiology of multiple sclerosis (MS) remains unknown, but its hallmark is the presence of demyelinating lesions in the central nervous system (CNS)[1,2]. Relapsing remitting MS is thought to result from an autoimmune attack on myelin antigen-bearing cells although the role of the immune system in progressive MS is less well defined[3–5]. Treatments for MS have primarily focused on limiting the immune attack on CNS cells to contain damage. While there have been several therapies with varying degrees of efficacy in slowing disease, reparative therapies have remained elusive. A relatively recent area of investigation has focused on myelin repair, which could provide an alternative or additional approach to therapeutic options currently available.

During the evolution of an MS lesion, oligodendrocytes are lost to varying degrees, and demyelinated axons may undergo secondary degeneration. The presence of demyelinated but intact axons and thinly myelinated axons, which are thought to be partially remyelinated, suggests that the opportunity for remyelination exists even in adults with MS[6,7]. Remyelination likely depends on the mobilization, migration, and maturation of oligodendrocyte progenitor cells (OPCs) into mature myelin-producing oligodendrocytes (OLs)[8,9]. The discovery that OPCs were present in adults at much higher quantities than previously thought provided substantial support to the rationale for developing therapies based on OPC stimulation[10–14]. OPC maturation and ultimately myelin production involve a number of distinct steps and pathways[15,16], and a greater understanding of these is critical to developing novel approaches to repair[7].

Several studies have been conducted both dissecting the pathways involved in the maturation of OPCs in vivo and targeting the process to stimulate repair. One molecule that has been extensively studied is the thyroid hormone, triiodothyronine (T3), which triggers a maturation process in which OPCs stop proliferating and differentiate into myelin producing cells[17]. Previous animal studies have demonstrated that administration of T3 improved remyelination, indicating a critical role of this molecule[18,19].

While the proteins of myelin have been a focus of the immunologic aspect of MS, myelin is comprised primarily of lipids, and one histologic feature of mature OLs is in fact, lipid-laden vacuoles. Thus, in the production of new myelin, significant lipid biogenesis, cholesterol in particular, must be dramatically increased[20]. Cholesterol blocking studies have shown mixed results but suggest that major depletion of cholesterol is deleterious to OL lineage cells[21–24]. Furthermore, several differentiation enhancing compounds have uncovered cholesterol biosynthesis as being an important signaling pathway[25–27].

Quetiapine is an atypical antipsychotic that is used in several different psychiatric settings. It is also being investigated as a possible therapeutic agent for enhancing remyelination in MS based on positive data *in vitro* [28–30] and *in vivo*. In animal models of demyelination, quetiapine ameliorates EAE, likely at least partially through its effects on the immune system[31], as well as promotes OPC differentiation in cuprizone models[28,32–34].

To further elucidate the mechanisms by which agents such as T3 and quetiapine produce their beneficial effects on OPC differentiation, we sought to dissect signaling pathways that promote differentiation, thereby determining whether OPC differentiation and remyelination could be enhanced through targeting complementary and additive pathways.

## Materials and methods

### Oligodendrocyte progenitor cells cultures

OPCs were obtained from cerebral cortices of P4-P7 rodent pups as described previously [35]. Rat pups were decapitated then cortices were dissected and enzymatically dissociated using Neural Dissociation Kit (P) (Miltenyi Biotec). OPCs were positively selected by labeling with A2B5 microbeads and passing through magnetic columns (Miltenyi Biotec). The cells were plated and expanded over 3–4 days in OPC media (modified from [36], composed of Dulbecco’s Modified Eagle Medium with B27, glutamine, penicillin/streptomycin, sodium pyruvate, trace elements B (all from ThermoFisher Scientific), apo-transferrin 100 μg/mL, bovine serum albumin 100 μg/mL, progesterone 60 ng/mL, putrescine 16 μg/mL, sodium selenite 40 ng/mL, insulin 50 μg/mL insulin, N-acetyl cysteine 5 μg/mL, biotin 10 ng/mL, hydrocortisone 50 ng/mL (all from MilliporeSigma)) with recombinant human PDGF-AA (PeproTech) until an optimal density was reached. Following proliferation, cells were differentiated over 96 hours in OPC media supplemented with either T3 (45nM; Sigma-Aldrich), Quetiapine Hemifumarate (1μM, Sigma-Aldrich), or both. Media was replenished at 48 hours. To inhibit cholesterol synthesis Betulin (0.03, 0.3 and 3μg/mL; Sigma-Aldrich) or Simvastatin (0.1, 1 and 10μM; Sigma-Aldrich) was added to the media at the beginning and 48 hours later during the differentiation assay. All the compounds were diluted in DMSO at a final concentration of 0.1% of the final volume. 0.1% DMSO was used as a vehicle control.

For the experiments in which the B27 supplement was not used, it was replaced by differentiation cocktail excluding T3, but including all other constituents of B27 supplement; L-Carnitine 2μg/ml, Ethanolamine 1μg/ml, D-galactose 15μg/ml, Putrescine 16.1μg/ml, biotin 10 ng/mL, Sodium Selenite 14.35ng/ml, Corticosterone 20ng/ml, Linoleic acid 1μg/ml, Linolenic acid 1μg/ml, Lipoic acid 47ng/ml, Progesterone 6.3ng/ml, Retinol acetate 100 ng/ml, Retinol (all trans) 100 ng/ml, D,L-alpha-Tocopherol 1μg/ml, D,L–alpha-Tocopherol acetate 1μg/ml, Albumin (bovine) 2.5mg/ml, Catalase 2.5μg/ml, Glutathione 1.0 μg/ml, Insulin 4 μg/ml, Superoxidase dismutase 2.5 μg/ml, Transferrin 5 μg/ml all from MilliporeSigma.

### Quantitative PCR

RNA was isolated from cultured OPCs using RNeasy Plus Mini Kit (Qiagen). Then cDNA was synthesized from the isolated mRNA using iScript cDNA Synthesis Kit (Bio-Rad). Quantitative PCR was carried out on these samples using SensiMix SYBR & Fluorescein Kit (Bioline) in the CFX384 Touch Real-Time PCR Detection System (Bio-Rad). Targets were normalized to the *hprt1* reference gene and delta-delta CT analysis was performed to determine the fold change in expression of each gene. Target genes and their sequences were: *mbp* Forward 5’-3’ CACAAGAACTACCCACTACGG, Reverse 5’-3’ GCCTCTCCCCTTTCCTTG, *hmgcs1* Forward 5’-3’ GATGGTGTAGATGCTGGAAAGTA, Reverse 5’-3’ GTCAGGCAGAGAGAGTTGATG, *hmgcr1* Forward 5’-3’ AAGAGTCGCTGTGTTCATCTC, Reverse 5’-3’ CCTGCTTGTACTCTGCTCTAAC, *fdft1* Forward 5’-3’ ACTGGCACTTCCCTACTAGA, Reverse 5’-3’ CGTAGCCTACTAACCACCAATAC, *sqle* Forward 5’-3’ TGCAGTCTATGCCACGTATTT, Reverse 5’-3’ AGAGCACGCTTTGTACAGTATAG, *cyp51* Forward 5’-3’ ACTGAAAGACTCCTGGGTAGA, Reverse 5’-3’ CAAACGGCACATAGGCAAAC, *hprt1* Forward 5’-3’ GGTGAAAAGGACCTCTCGAAG, Reverse 5’-3’ GCTTTTCCACTTTCGCTGATG.

### Immunocytochemistry

The differentiated OPCs were fixed with 4% PFA for 15 minutes. After fixation they were incubated in mouse monoclonal anti myelin basic protein (MBP) (1:1000; Biolegend clone SMI-99) and rabbit polyclonal anti-Olig2 (1:1000; MilliporeSigma) antibodies overnight. The next day they were incubated in secondary antibodies anti-mouse Alexa Fluor 488 (1:1000; Invitrogen) and anti-rabbit 594 (1:1000; Invitrogen) for 2 hours. DAPI nuclear staining was performed for 10 minutes which was washed prior to imaging. Images were taken using IncuCyte S3 50400, analysis was performed using IncuCyte S3 2017A Rev2 Version. Total integrated intensity for MBP (total sum of the objects’ fluorescent intensity in the image: Fluorescent units x μm^2^/image) and the number of Olig2 positive cells was quantified, and a ratio of MBP integrated intensity/Olig 2 number was determined for 16 images per well, from 2 wells per condition, in a total of 3 experiments. Higher resolution images were taken using an epifluorescence microscope at 20x and laser confocal microscope using 63x objectives. For cholesterol staining, after fixation the cells were stained with mouse monoclonal anti-Olig2 (1:500; MilliporeSigma) overnight. The next day they were incubated with secondary anti-mouse 594 (1:1000; Invitrogen) and filipin complex (MilliporeSigma) 50μg/ml for 2 hours. Images were taken using a Keyence BZ-X700 microscope. Analysis was performed using ImageJ v 1.52a National Institute of Health. Filipin stained area in pixels and number of Olig2 positive cells was quantified, and a ratio of filipin stained area/Olig2 number was determined for 6 images per slide, from 3 slides per condition.

### Live cell imaging

Cytotoxicity of the different compounds was determined by culturing OPCs in the presence of compound and IncuCyte Cytotox Green Reagent (Essen Bioscience) in OPC media. In cells where the membrane integrity had been affected the reagent binds to DNA, increasing its fluorescence. Nine images were taken per well every 2 hours over a 72 hour period in IncuCyte S3 50400. There were 2 wells per replicate. Analysis was done using IncuCyte S3 software, version 2017A Rev2.

### Gene array analysis

Afflymetrix microarrays were completed on T3, Quetiapine, T3 plus Quetiapine and control OPC cultures at baseline (Day 0), 48 hours and 96 hours after differentiation assay was initiated. Gene set enrichment analysis (GSEA)[37] was done comparing T3 treated samples to Quetiapine and T3 plus Quetiapine treated samples, to determine pathway enrichment for genes associated with T3, QTP, and T3+QTP vs. control[38].

### Western blot

OPCs in culture were lysed in RIPA buffer in the presence of protease and phosphatase inhibitors (Halt Protease and Phosphatase Inhibitor Cocktail; ThermoFisher) following 96 hours of differentiation in the presence of T3 45nM, Quetiapine 1μM, combination of both or DMSO vehicle. The protein was quantified using a BCA assay and samples were denatured by heating to 95° C for 10 minutes with 4X SDS reducing buffer (Boston BioProducts). Electrophoresis was performed in a 12% polyacrylamide gel for 1hr 15 mins at 110V, and separated proteins were transferred to a nitrocellulose membrane using Trans-Blot^®^ Turbo^™^ blotting system (Bio-Rad). After blocking with 5% powder milk, membranes were stained with MBP (1:1000) (clone SMI99, Biolegend) and actin (1:5000) (Sigma-Aldrich, clone AC-74) antibodies overnight, then with secondary antibodies IRDye^®^ 700CW and 800CW for detection using Odyssey imaging system (LI-COR). Intensity was determined for MBP and Actin bands. An MBP/Actin ratio was determined for each sample then normalized to the control condition. A total of six independent experiments were performed. Each set of samples was run in duplicate. The mean of the duplicates was included in the analysis shown.

### Animals

All animal protocols were approved and adhered to the guidelines of Johns Hopkins Institutional Animal Care and Use Committee. Rats were maintained in a pathogen-free facility at Johns Hopkins University. SAS Sprague Dawley timed-pregnant rats were purchased from Charles River.

### Statistical analysis

For qPCR and immunohistochemistry, one-way ANOVA with Tukey’s multiple comparison test was used. Bars represent standard error of the mean. Student’s t-test for western blot analysis, comparisons between each treatment condition and control were performed. A p-value of ≤ 0.05 was used as the cutoff for significance for all statistical tests.

## Results

### Quetiapine and T3 have additive effects in inducing differentiation of OPCs

**Fig 1:**
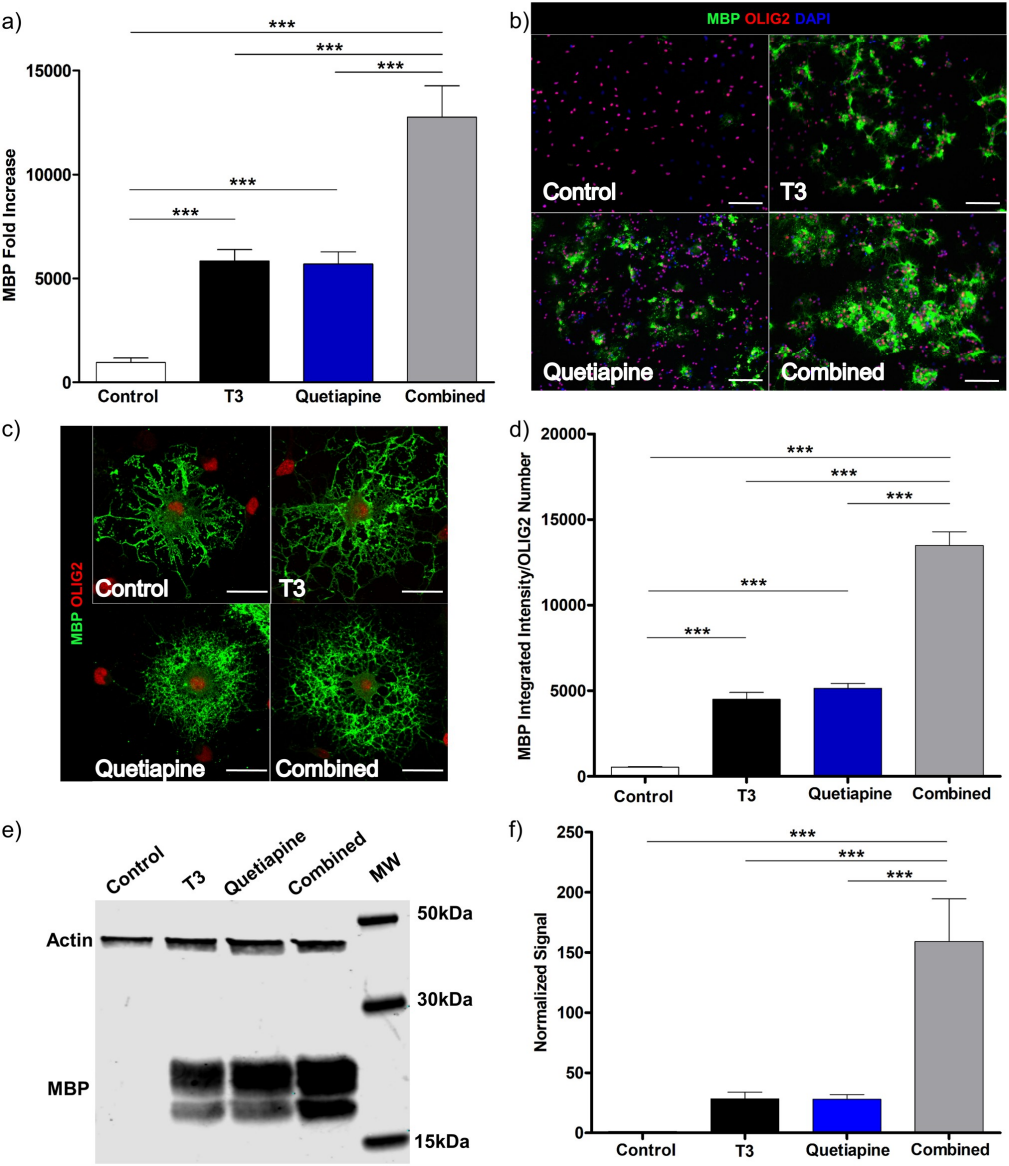
Quetiapine induces differentiation of OPC and has an additive effect on T3 induced differentiation. OPCs isolated from 4–7 days old rats were cultured for 96 hrs under PDGF 20ng/ml, were then induced to differentiate in OPC media with the addition of T3 45nM (Black), Quetiapine 1μM (Blue), or both (Grey) for 96 hrs. OPC media with 0.1%DMSO (vehicle) was used as control (White). **(a)** MBP expression was measured by qPCR. A pre-treatment day 0 sample was used to normalize gene results. Error bars represent standard error of the mean from 3 independent isolations and experiments. One-way ANOVA analysis with Tukey’s multiple comparison analysis was run (p **<0.001, *** p< 0.0001). **(b)** Immunocytochemistry: Plated cells were fixed with 4% PFA, stained for MBP (Green), Olig2 (Red) and DAPI (Blue). Representative images at 20X, for Control (upper left), T3 (upper right), Quetiapine (lower left), T3 plus Quetiapine (lower right), scale bar 100μm. **(c)** Laser scanning confocal microscopy images 63X of Control (upper left), T3 (upper right), Quetiapine (lower left), T3 plus Quetiapine (lower right), scale bar 20 μm. **(d)** A ratio was determined between MBP intensity quantification and OLIG2 positive cell number, the bar graph represents the results from 6 different experiments and the error bars are the standard error of the mean. One-way ANOVA analysis with Tukey’s multiple comparison analysis was run (*** p< 0.0001). **(d)** Protein lysate from OPC culture was obtained after 4 days of treatment with the conditions mentioned before, and western blot was performed, **(e)** representative image and **(f)** quantification results from 6 different experiments, one-way ANOVA analysis with Tukey’s multiple comparison analysis was run (*** p< 0.0001).

To investigate the effects of quetiapine and T3 on OPC differentiation, we generated primary OPC cultures, added each agent alone or in combination and conducted a comprehensive evaluation of the characteristic features of OPC differentiation. Specifically, we utilized genetic, protein, and morphological analyses to quantify the differentiation process. The expression and production of myelin basic protein was evaluated by qPCR and IC from cultures treated with either T3, quetiapine, or both. As shown in Fig 1a, there was a significant increase in MBP gene expression with either agent, alone and when tested in combination (in every case p<0.001). When combined their effect on MBP expression was additive. These effects are not observed at earlier stages of the experiment (S1 Fig). In order to determine that the effects of quetiapine were not induced by the small amount of T3 found in the B27 media supplement, the specific components of the media were added individually in place of the B27 supplement, the results of these experiments showed that quetiapine induces OPC differentiation, even in the absence of T3 (S2a Fig). The small amount of T3 in the commercial B27 supplement did result in higher MBP expression than in the home made media with no T3 (S2b Fig), which reached significance in some but not all comparisons. Next, we evaluated T3 and quetiapine’s effect on MBP surface expression using IC (Fig 1b). As shown in Fig 1c, all cells showed a characteristic OL morphology and have MBP expression but quetiapine treated cells have a round shaped halo surrounding the nuclei (bottom panels), whereas T3 only treated cells (top right panel) appear to have longer and more branched projections. While each agent led to an increase in MBP expression, the combination was additive (Fig 1d). To further quantify the protein expression, we harvested lysates from the cultures and did a Western blot for MBP. These showed an increase in MBP synthesis of each compound alone and an additive effect of the combination of T3 and quetiapine, (Fig 1e and 1f).

### Quetiapine induces cholesterol biosynthesis pathway genes expression with and without T3

**Fig 2:**
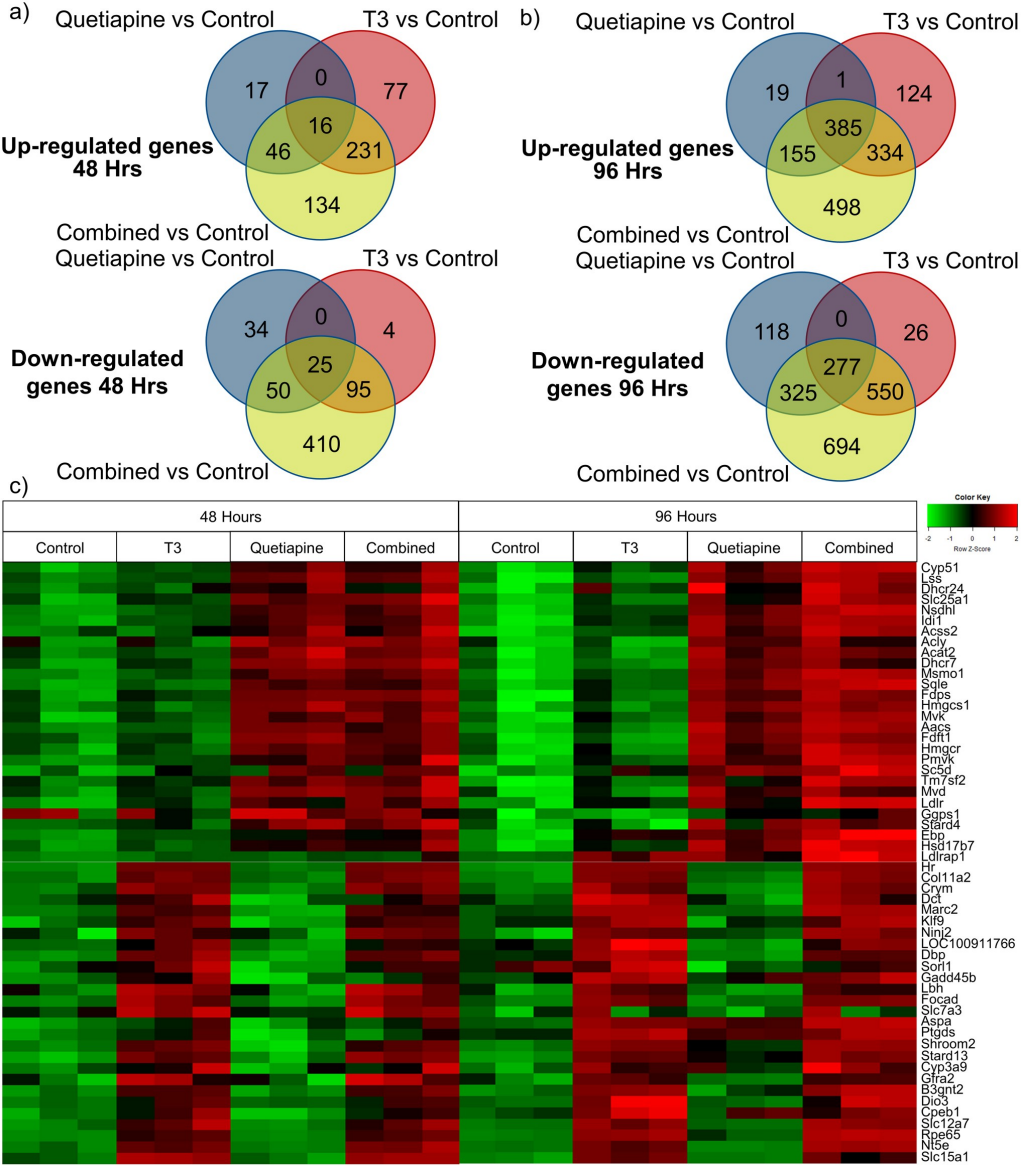
Gene expression array showing patterns of differentially regulated genes in OPCs treated with either T3 or quetiapine at 48 and 96 hours of culture. Affymetrix gene array was performed on OPCs treated with; T3 45nM, Quetiapine 1μM, both, or vehicle control for 48 hrs and 96 hrs. Results were normalized to day 0 pre-treatment samples. Venn diagrams showing numbers of upregulated (top) and downregulated (bottom) genes at 48 hrs **(a)** and 96 hrs **(b)** for T3, Quetiapine and T3 plus Quetiapine conditions compared to control condition. Significance was defined as fold change >1.5 and false discovery rate < 0.05. **(c)** Heat map showing patterns of genes at 48 hours and 96 hours upregulated by Quetiapine (top half) and T3 (bottom half).

To determine the signaling pathways that each of these agents mediated its individual effects through, we conducted a gene array to identify differentially modulated genes. To achieve this (as seen in Fig 1) OPC cultures were established, treated with either T3, quetiapine or both, and differential patterns of gene expression were analyzed. Interestingly, there were distinct patterns of effects of upregulation and downregulation on gene expression by the agents, as depicted in the Venn diagrams (Fig 2a and 2b). These distinct patterns are further illustrated in a heat map showing the top genes differentially upregulated by both compounds (Fig 2c). A full list of genes significantly upregulated by T3 (S1 Table), quetiapine (S2 Table), or combination therapy (S3 Table) at both 48 and 96 hours, is also provided. Further validating our findings, several genes previously found to be upregulated by T3 (Klf9, Hr, Dbp, and Nt5e) are also upregulated by T3 in our data [17].

**Fig 3:**
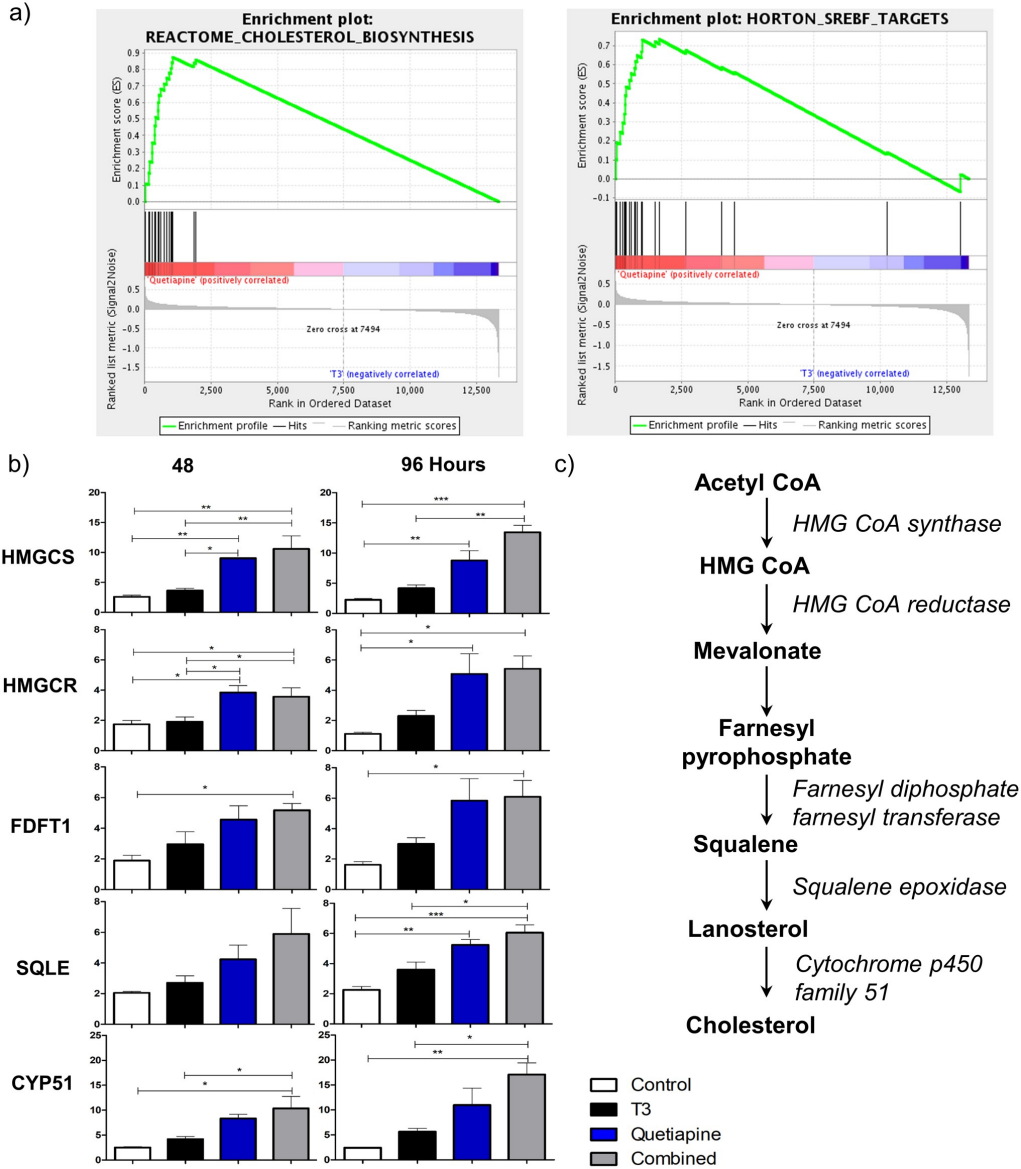
Gene set enrichment analyses and qPCR reveal cholesterol biosynthesis genes being more potently induced by quetiapine than T3. **(a)** Enrichment plots for cholesterol pathway genes sets that were differentially upregulated by quetiapine compared to T3. **(b)** qPCR for *hmgcs1*, *hmgcr*, *fdft1*, *sqle*, and *cyp51* was done using RNA isolated from OPCs induced to differentiate in OPC media with the addition of T3 45nM (Black), Quetiapine 1μM (Blue), or both (Grey) at 48 and 96 hrs. OPC media with 0.1%DMSO was used as control (White). A pre-treatment day 0 sample was used to normalize gene results. Error bars represent standard error of the mean from 3 independent isolations and experiments. (* p<0.01, ** p< 0.001, *** p< 0.0001). **(c)** Schematic representation of cholesterol synthesis pathway, intermediary products are represented in **bold** letters, enzymes involved in *italics*.

To determine the signaling pathways induced by quetiapine we performed a gene set enrichment analysis (GSEA), which shows cholesterol synthesis pathway gene sets upregulated on quetiapine treated samples compared to T3 (Fig 3a). Accordingly, within these gene sets, we found that the genes involved in sequential steps of the cholesterol synthesis pathway were upregulated (Fig 3b and 3c), such as *hmgcs1*, *hmgcr*, *fdft1*, *sqle*, cyp51 by qPCR analysis. This increase seems to be more completely mediated by quetiapine because each one of these genes was significantly up regulated in quetiapine alone and the combination but not with the T3 alone condition. This also confirmed the Affymetrix gene chip results, which show a general increase in gene expression from each step of the cholesterol synthesis pathway. Hallmark Pathway analysis identified E2F targets and G2M checkpoint gene sets as also being modulated by each compound individually as well as the combination of T3 and quetiapine (S4 Table).

**Fig 4:**
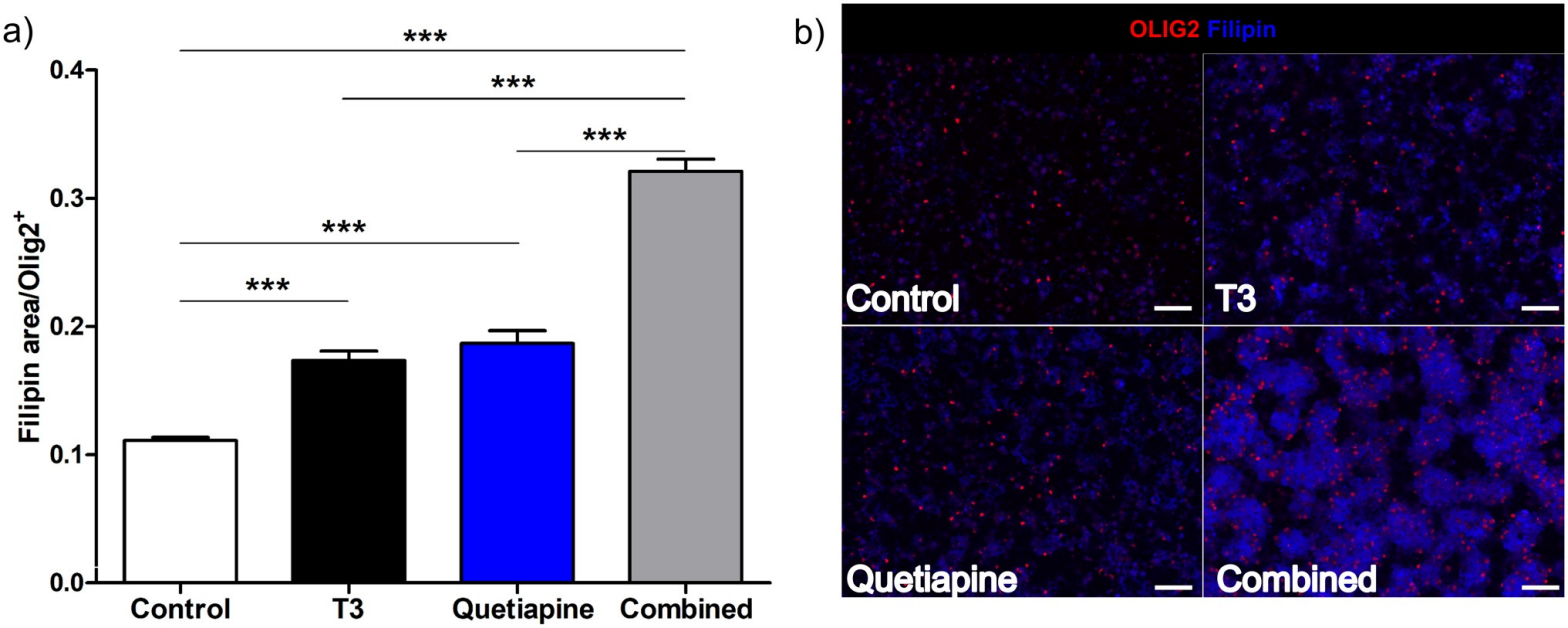
T3 and Quetiapine induce cholesterol production. OPCs isolated from 4–7 days old rats and cultured for 96 hrs under PDGF 20ng/ml, were then induced to differentiate in OPC media with the addition of T3 45nM (Black), Quetiapine 1μM (Blue), or both (Grey) for 96 hrs. OPC media with 0.1%DMSO (vehicle) was used as control (White). **(a)** Cells were fixed with 4% PFA then stained for Olig2 and Filipin. A ratio between filipin stained area and OLIG2 positive cell number was determined, the bar graph represents the results from 6 different experiments and the error bars are the standard error of the mean. One-way ANOVA analysis with Tukey’s multiple comparison analysis was run (*** p< 0.0001). **(b)** Representative images at 20X, for Control (upper left), T3 (upper right), Quetiapine (lower left), T3 plus Quetiapine (lower right), scale bar 200μm.

In order to confirm that the expression of cholesterol genes was associated with an increase in cholesterol content of the cells, a staining for cholesterol was performed. It showed an increase in cholesterol in the OPCs treated with T3 and quetiapine, and an additive effect with both compounds (Fig 4).

### Cholesterol production inhibitors betulin and simvastatin suppress MBP gene expression and protein production without affecting cell viability

**Fig 5:**
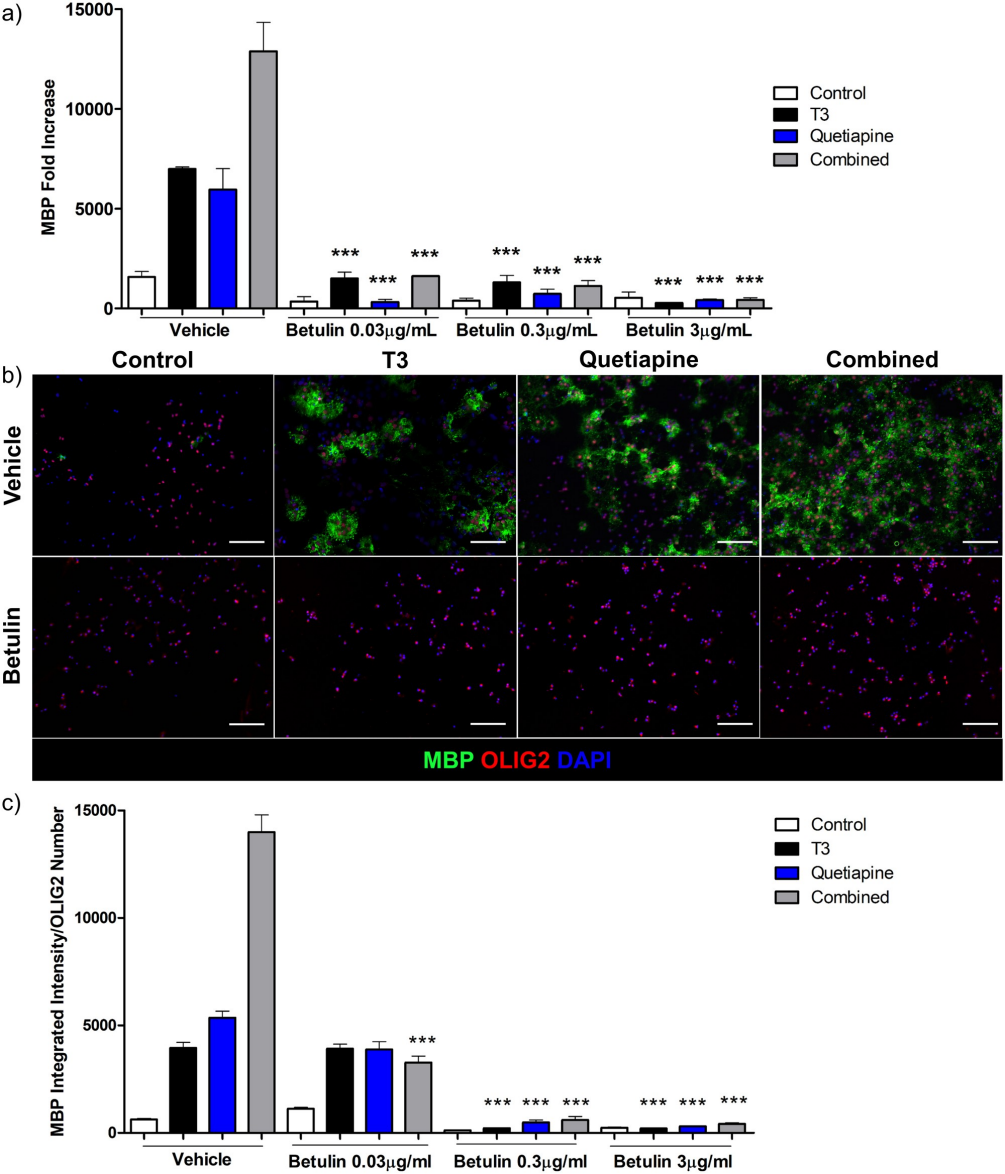
Cholesterol synthesis inhibitor betulin suppresses OPC gene and protein expression of MBP. **(a)** qPCR for MBP was performed using RNA isolated from OPCs treated with T3 45nM (Black), Quetiapine 1μM (Blue), or both (Grey) for 96 hours or OPC media with 0.1%DMSO (White), in presence of different doses of betulin 0.03, 0.3 and 3μg/ml during the same time. A pre-treatment day 0 sample was used to normalize gene results. Error bars represent standard error of the mean from 3 independent isolations and experiments. One-way ANOVA analysis with Tukey’s multiple comparison analysis was run (*** p < 0.0001 for betulin treated conditions compared to same condition without betulin e.g. T3 with betulin 3μg/ml vs T3 vehicle). **(b)** Immunocytochemistry for MBP (Green), Olig2 (Red) and DAPI (Blue) from OPCs differentiated with vehicle (upper row) and betulin 3μg/ml (lower row). **(c)** A ratio was determined between MBP intensity quantification and OLIG2 positive cell number, from 3 experiments with OPCs treated with T3 45nM (Black), Quetiapine 1μM (Blue), or both (Grey) for 96 hrs or OPC media with 0.1% DMSO (White), in presence of different doses of betulin 0.03, 0.3 and 3μg/ml during the same time. Error bars represent standard error of the mean. One-way ANOVA analysis with Tukey’s multiple comparison analysis was run (*** p ≤ 0.0001 for betulin treated conditions compared to same condition without betulin).

To further investigate the specific role of cholesterol synthesis, we tested the effect of the cholesterol synthesis inhibitor, betulin, on MBP expression. Betulin inhibits cholesterol synthesis at the first step of its biosynthesis by interfering with the activation of the sterol regulatory element-binding protein (SREBP)[39]. Cultures were generated as above, with the addition of T3, quetiapine, or both, with or without betulin at different concentrations, and MBP gene expression was quantified (Fig 5a). In all cases betulin effectively shut off MBP gene expression. Immunostaining for MBP revealed similar results (Fig 5b and 5c), MBP production was all but eliminated, which again supports the contention that differentiation has been halted due to the impairment of cholesterol synthesis, and not due to cell death as described below (S3 Fig).

**Fig 6:**
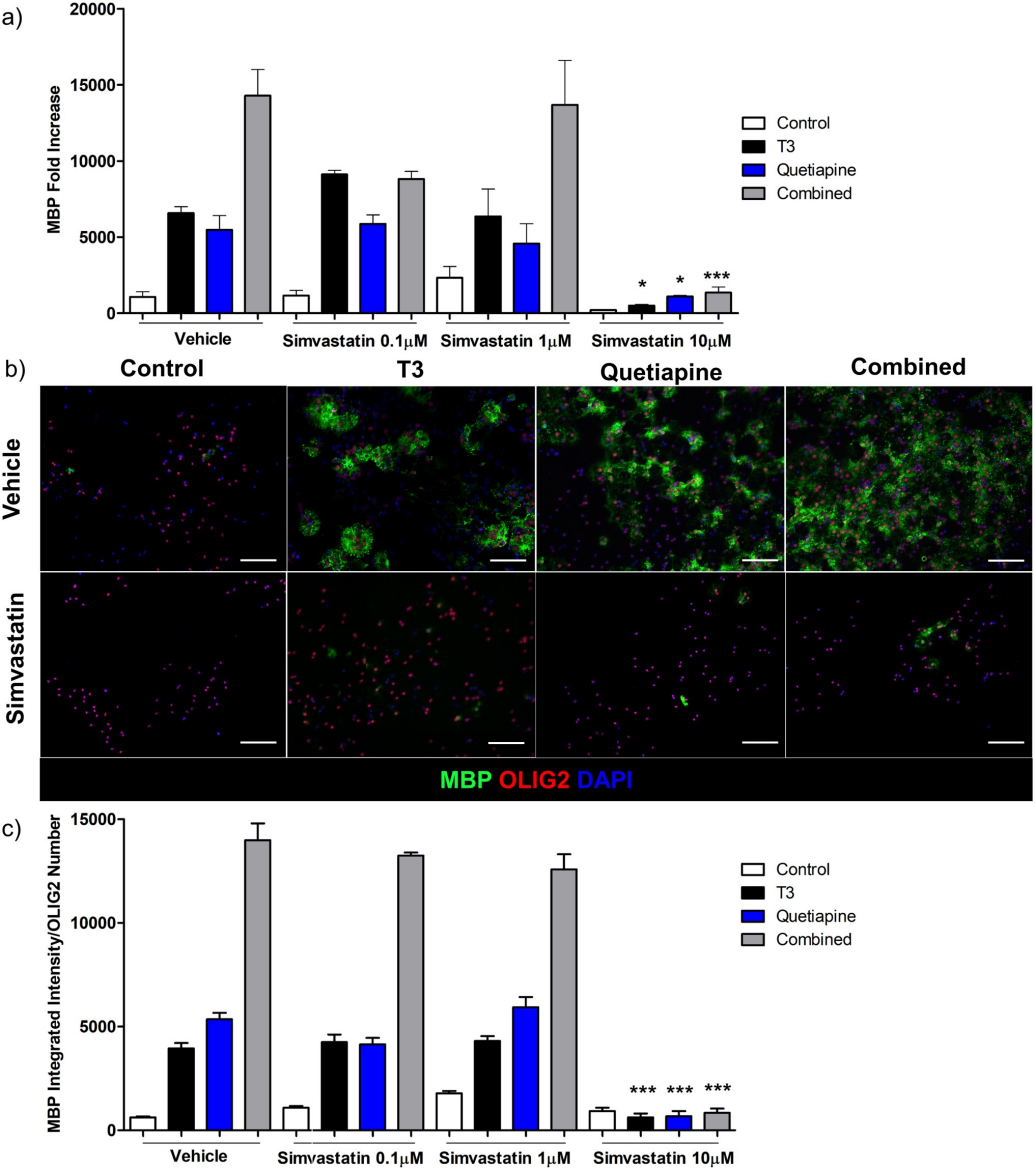
Cholesterol synthesis inhibitor simvastatin suppress OPC gene and protein expression of MBP. **(a)** qPCR for MBP was performed using RNA isolated from OPCs treated with T3 45nM (Black), Quetiapine 1μM (Blue), or both (Grey) for 96 hrs or OPC media with 0.1%DMSO (White), in presence of different doses of simvastatin 0.1, 1 and 10μM during the same time. A pre-treatment day 0 sample was used to normalize gene results. Error bars represent standard error of the mean from 3 independent isolations and experiments. One-way ANOVA analysis with Tukey’s multiple comparison analysis was run (*** p < 0.0001 for simvastatin treated conditions compared to same condition without simvastatin e.g. T3 with simvastatin 10μM vs T3 vehicle). **(b)** Immunocytochemistry for MBP (Green), Olig2 (Red) and DAPI (Blue) from OPCs differentiated with vehicle (upper row, same representative images as in Fig 5b) and simvastatin 10μM (lower row). **(c)** A ratio was determined between MBP intensity quantification and OLIG2 positive cell number, from 3 experiments with vehicle (same vehicle conditions data as in 5c since these experiments were performed at the same time) and OPCs treated with T3 45nM (Black), Quetiapine 1μM (Blue), or both (Grey) for 96 hrs or OPC media with 0.1%DMSO (White), in presence of different doses of simvastatin 0.1, 1 and 10μM during the same time. Error bars represent standard error pf the mean. One-way ANOVA analysis with Tukey’s multiple comparison analysis was run (*** p < 0.0001 for simvastatin treated conditions compared to same condition without simvastatin).

Similarly, simvastatin which inhibits cholesterol biosynthesis downstream of SREBP by blocking HMG-CoA reductase[40], then cholesterol biosynthesis, also reduced MBP gene expression (Fig 6a) and protein levels (Fig 6b and 6c) in OPCs but only at the highest concentration studied.

In addition to DAPI staining indicating that cells were still present, we both enumerated OLIG2 positive and DAPI positive cells, and measured cell survival longitudinally using IncuCyte to assure that the inhibitory effects were not a result of toxicity by betulin or simvastatin (S3 and S4 Figs). Neither betulin nor simvastatin resulted in significant cell death, or shifts in the ratios of OLIG2+/DAPI+cells of the cultures at the highest doses of betulin and simvastatin, which suggests that the inhibition resulted from targeting a specific pathway[39] rather than the known capacity of betulin to induce apoptosis at higher concentrations [41]. These data further support a critical role of cholesterol biosynthesis in MBP gene expression and production during the process of OPC differentiation.

## Discussion

The identification of signaling pathways involved in OPC differentiation, which may facilitate myelin repair, has become the focus of both biological studies and therapy development [6,7,15–18,25–27,30]. As it becomes clearer that myelin regeneration is a plausible strategy for demyelinating diseases, a greater understanding of the mechanisms by which OPCs can be differentiated into mature, myelin-producing OLs will be critical for rational development of novel therapies. The progressive form of MS in particular suffers from a dearth of treatments and has remained essentially unimproved by immune-based therapies. Thus, a focus on developing novel approaches to targeting the neuroprotective/regenerative capacities of CNS cells is critical to advancing treatments.

While it is unclear how the damage to axons, neurons, and OLs is initiated and in some cases maintained, the potential for repair to damage seems promising, particularly in the early phases, in which axons may still be intact and amenable to remyelination. The presence of OPCs and numerous studies indicating that they can be mobilized and differentiated, even in adults, highlights this possibility, as the elements needed to repair may be already in place[1,8–11]. A growing number of reports have demonstrated that in animal models, following demyelination, OPCs are expanded, mobilized and differentiated, and remyelination of axons has been demonstrated, indicating a repair of myelin damage[11,32,33]. Thus, the potential for this process to produce a notable biological effect is high.

T3 is a well-studied agent that is known to induce OPC differentiation. Because of its potent effects, it has become a standard component of OL culture [17,18,26,30,42,43]. In addition to its *in vitro* effects, it has been shown to have a positive *in vivo* effect as demonstrated by its ability to promote developmental myelination mediated by OPCs [43] and to mitigate cuprizone-mediated demyelination [42]. In addition to confirming known signaling pathways enhanced by T3, such as KLF9, we report additional genes, some of which appear to work in concert with other pro-differentiation drugs, such as quetiapine. These results add to the understanding of the mechanisms by which T3 exerts its critical effects on OPCs.

Quetiapine is an intriguing agent that has been shown to have several possible therapeutic effects in the context of MS/EAE. An atypical antipsychotic, it has been shown to suppress autoimmunity, scavenge free radicals, inhibit inflammatory effects of microglia[44,45] and astrocytes, as well as protect the blood brain barrier after trauma[46]. Thus, it has appeal as a possible therapeutic agent with the fact is already licensed to be use as antipsychotic. In addition to these known effects, evidence suggests that it may have a distinct influence on lipid profiles, which led us to hypothesize that it may specifically regulate cholesterol synthesis[47] which is necessary for myelin formation[20], as has been shown with other atypical antipsychotic drugs[48].

Cholesterol biosynthesis is a complex process that is tightly regulated in mammals. In a multi-step process, isopentenyl pyrophosphate is formed in the mevalonate pathway, followed by formation of squalene by the condensation of six molecules of isopentenyl pyrophosphate, which then undergoes a cyclic reaction leading to reformation that produces cholesterol. Here we show that several enzymes critical to catalyzing the process, namely *hmgcs1*, *hmgcr*, *fdft1*, *cyp51*, *and sqle*, are induced by T3 and quetiapine or quetiapine alone. In combination, the treatments have an additive effect on their expression. To demonstrate functional significance, we sought to specifically block the process of cholesterol synthesis to test whether the pro-differentiation effects of T3 and quetiapine would be lost. Towards this end, we utilized the compound betulin, which is an inhibitor of sterol regulatory element-binding proteins (SREBPs) processing, a family of transcription factors that regulate the production of cholesterol, lipids, and fatty acids. SREBPs serve an essential role in the homeostasis of cholesterol production, and disruption of their function produces a profound effect on cholesterol synthesis[49]. We discovered that the addition of betulin abrogated the stimulatory effects of T3 and quetiapine, whether alone or in combination, which demonstrated that synthesis of cholesterol was critical to the induction of MBP expression by both of these agents.

Prior studies of statins have revealed potent anti-inflammatory effects of HMG-CoA reductase inhibitors via depletion of isoprenoids (farnesyl-pyrophosphate and geranylgeranyl-pyrophosphate) rather than cholesterol in immune cells[40,50–57]. However, its effects on myelination have been observed to be variable with some studies suggesting inhibitory effects and others suggesting no effect or even positive effects on OPCs through inhibition of Rho [24,58]. Our data is consistent with the notion that high doses of statins potentially limit cholesterol synthesis and myelin production in OL lineage cells[23,24,59], but low doses may still have beneficial effects on inflammation and other pathways without depleting cholesterol to the extent that OL lineage cells can no longer express and produce MBP.

While our study is consistent with prior reports of the importance of the cholesterol biosynthesis pathway signaling in OL lineage cell differentiation[20–23,27], it remains unclear how this lipid is critical for MBP gene expression and not just downstream formation of myelin, which is 70% lipid. The synthesis of isoprenoids, which are important for isoprenylation of certain cell signaling proteins and cell growth, plays a role in OL lineage differentiation, and blocking this step also could impair myelin formation[22]. Furthermore, we noticed a change in the morphology of the cells treated with quetiapine, characterized by the appearance of a halo around the nuclei, which could be explained be the spherical accumulation of cholesterol around the nuclei, but a complete understanding of this phenomenon will require further investigation of cholesterol transport.

It is worth noting that cholesterol synthesis is also involved in production of vitamin D, bile acids and hormones, all of which may be reduced in people with MS[60–69]. Therefore, studies aimed at understanding if there are intrinsic defects or inhibitors of the cholesterol biosynthesis pathway may be worthwhile. Further, given recent data that statins have anti-inflammatory effects both in the periphery and CNS, and thereby may inhibit brain atrophy in progressive MS[70], it will be critical to understand whether prolonged high concentrations of statins could possibly inhibit myelin repair by reducing cholesterol in OL lineage cells.

We note that our study differs in some respects from two other reports examining the effects of quetiapine on oligodendrocyte lineage cells [71,72]. Fang et al. showed induction of Olig1/2 by quetiapine. They utilized the CG4 cell line that expresses Olig1/2 upon differentiation. Our freshly isolated primary OPCs already expressed Olig 1/2 suggesting they are lineage committed and therefore may not need to further upregulate these transcription factors in order to differentiate into MBP expressing oligodendrocytes. Kondo et al. performed an ex vivo gene expression analysis of frontal cortex RNA from animals treated with quetiapine vs vehicle and found suppression of Cdkn 1a. While we did not see suppression of Cdkn1a in our array, other Cdkn family members were suppressed (see S3 Table) and this discrepancy may relate to timing of sampling as well as differential drug exposure *in vivo*.

Another potential limitation of our study is that some of the effects of T3 alone or combination therapy may have been masked by the low levels of T3 found in the commercial B27 supplement we used. Nonetheless, we were able to demonstrate the canonical T3 genes reported by others were induced by commercial B27 medium plus T3 as compared to medium alone. Most importantly, quetiapine still had the same pro-differentiating effects on OPCs even when used with homemade B27 excluding T3 supporting an independent role of this compound in mediating OPC differentiation through the cholesterol biosynthesis pathway.

In summary, this study reports the novel finding that quetiapine modulates the cholesterol biosynthesis pathway in an additive manner with T3, enhancing effects on OPC differentiation and myelin production. Taken together these results suggest the possibility that combinatorial approaches to induction of OPC differentiation may be useful. Further understanding of the signaling cues needed to promote myelin repair as well as those existing factors that inhibit OPC differentiation will be critical to translating these observations to a clinical setting.

## Supporting information

S1 Fig MBP expression is not significantly increased by T3 or Quetiapine at 48 hours. OPCs isolated from 4–7 days old rats were cultured for 96 hrs under PDGF 20ng/ml, then induced to differentiate in OPC media with the addition of T3 45nM (Black), Quetiapine 1μM (Blue), or both (Grey) for 48 hrs. OPC media with 0.1%DMSO (vehicle) was used as control (White). MBP expression was measured by qPCR. A pre-treatment day 0 sample was used to normalize gene results. Error bars represent standard error of the mean from 3 independent isolations and experiments. One-way ANOVA analysis with Tukey’s multiple comparison analysis was run (n.s. non-significant).
(TIF)

S2 Fig Quetiapine induces differentiation of OPC in the absence of T3. OPCs isolated from 4–7 day old rats were cultured for 96 hours with PDGF 20ng/ml in OPC media made with all of the components of B27 except T3. (**a**) After 96 hours, either T3 45nM (Black), Quetiapine 1μM (Blue), or both (Grey) was added to the media for an additional 96 hrs. OPC media with 0.1%DMSO (vehicle) was used as control (White). MBP expression was measured by qPCR. A pre-treatment day 0 sample was used to normalize gene results. Error bars represent standard error of the mean from 2 independent isolations and experiments. One-way ANOVA analysis with Tukey’s multiple comparison analysis was run (*** p< 0.0001). (**b**) Comparison between OPCs treated in media with either B27 (Red) supplement (as in Fig 1a) or with all of the components of B27 except T3 (White) (as in S2a Fig). MBP expression was measured by qPCR. A pre-treatment day 0 sample was used to normalize gene results. Significance was determined using a One-way ANOVA analysis with Tukey’s multiple comparison analysis (n.s. non significant, ** p< 0.001).
(TIF)

S3 Fig Cell toxicity over 48 hours. Cell toxicity assays of each compound were performed on cultured OPCs in the presence of IncuCyte Cytotox Green Reagent. **(a)** Ratio of number of dead cells at each time point by number of dead cells at the beginning of the experiment were calculated. Error bars represent standard error of the mean. 2-way ANOVA analysis with Tukey’s post tests were run (*** p < 0.0001 for stausporin compared to vehicle DMSO 0.1%).
(TIF)

S4 Fig OLIG2/DAPI ratio. OLIG2 and DAPI positive cells were enumerated and the ratio is shown for each condition, T3 45nM (Black), Quetiapine 1μM (Blue), or both (Grey) for 96 hrs. OPC media with 0.1%DMSO (vehicle) was used as control (White) in the presence or absence of betulin 3 μg/ml. Error bars represent standard error of the mean. A one-way ANOVA analysis showed no significant difference.
(TIF)

S1 Table Gene array T3 regulated genes. (XLSX)

S2 Table Gene array quetiapine regulated genes. (XLSX)

S3 Table Gene array T3 plus quetiapine regulated genes. (XLSX)

S4 Table Hallmark pathways regulated by T3, quetiapine, or combination. (XLSX)
