# Supplementary figures and images for: Quetiapine has an additive effect to triiodothyronine in inducing differentiation of oligodendrocyte precursor cells through induction of cholesterol biosynthesis
Source: PLoS One. 2019 Sep 6;14(9):e0221747. doi: 10.1371/journal.pone.0221747 (PMC6730995; doi:10.1371/journal.pone.0221747)

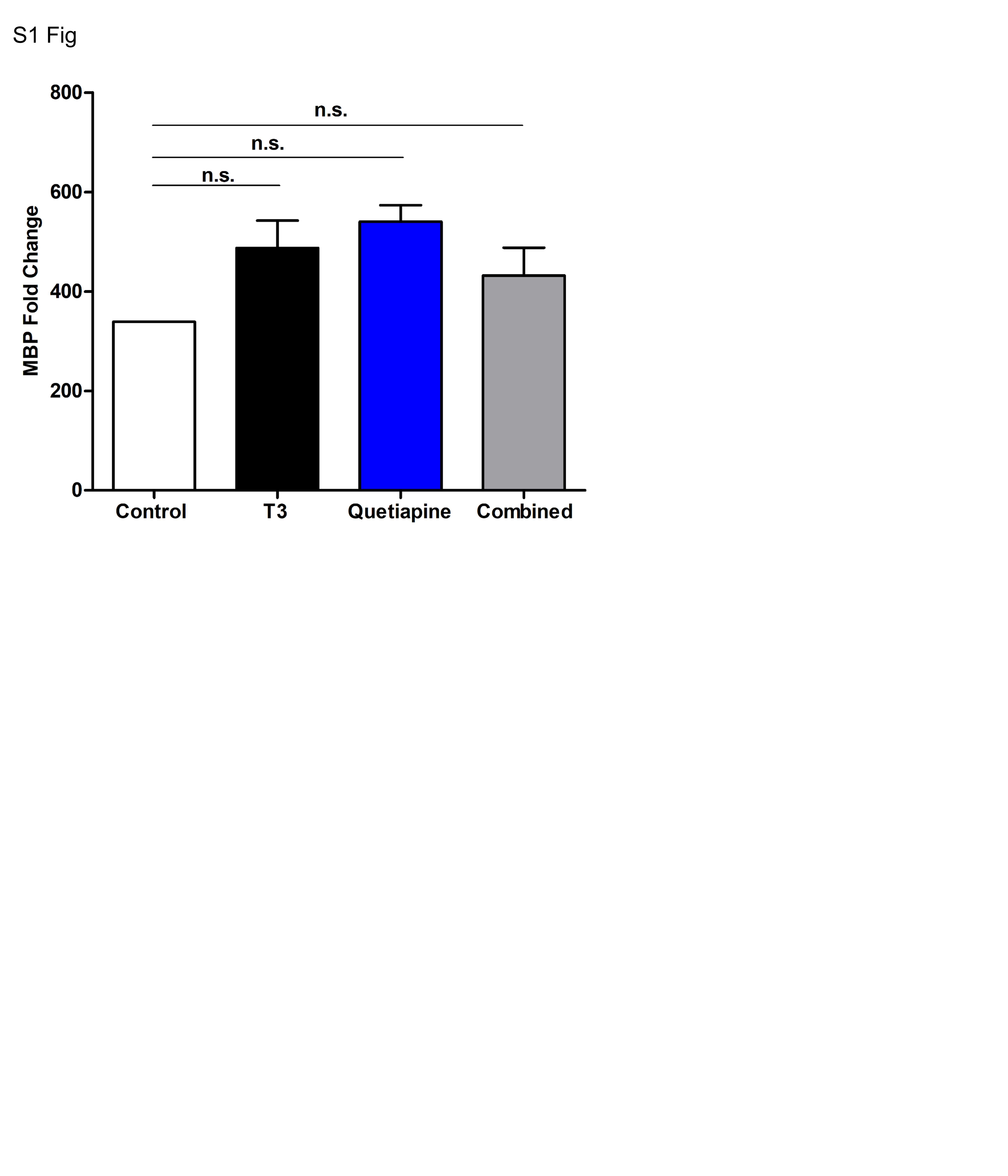

Supplement: S1 Fig — OPCs isolated from 4–7 days old rats were cultured for 96 hrs under PDGF 20ng/ml, then induced to differentiate in OPC media with the addition of T3 45nM (Black), Quetiapine 1μM (Blue), or both (Grey) for 48 hrs. OPC media with 0.1%DMSO (vehicle) was used as control (White). MBP expression was measured by qPCR. A pre-treatment day 0 sample was used to normalize gene results. Error bars represent standard error of the mean from 3 independent isolations and experiments. One-way ANOVA analysis with Tukey’s multiple comparison analysis was run (n.s. non-significant). (TIF) [file pone.0221747.s001.TIF]

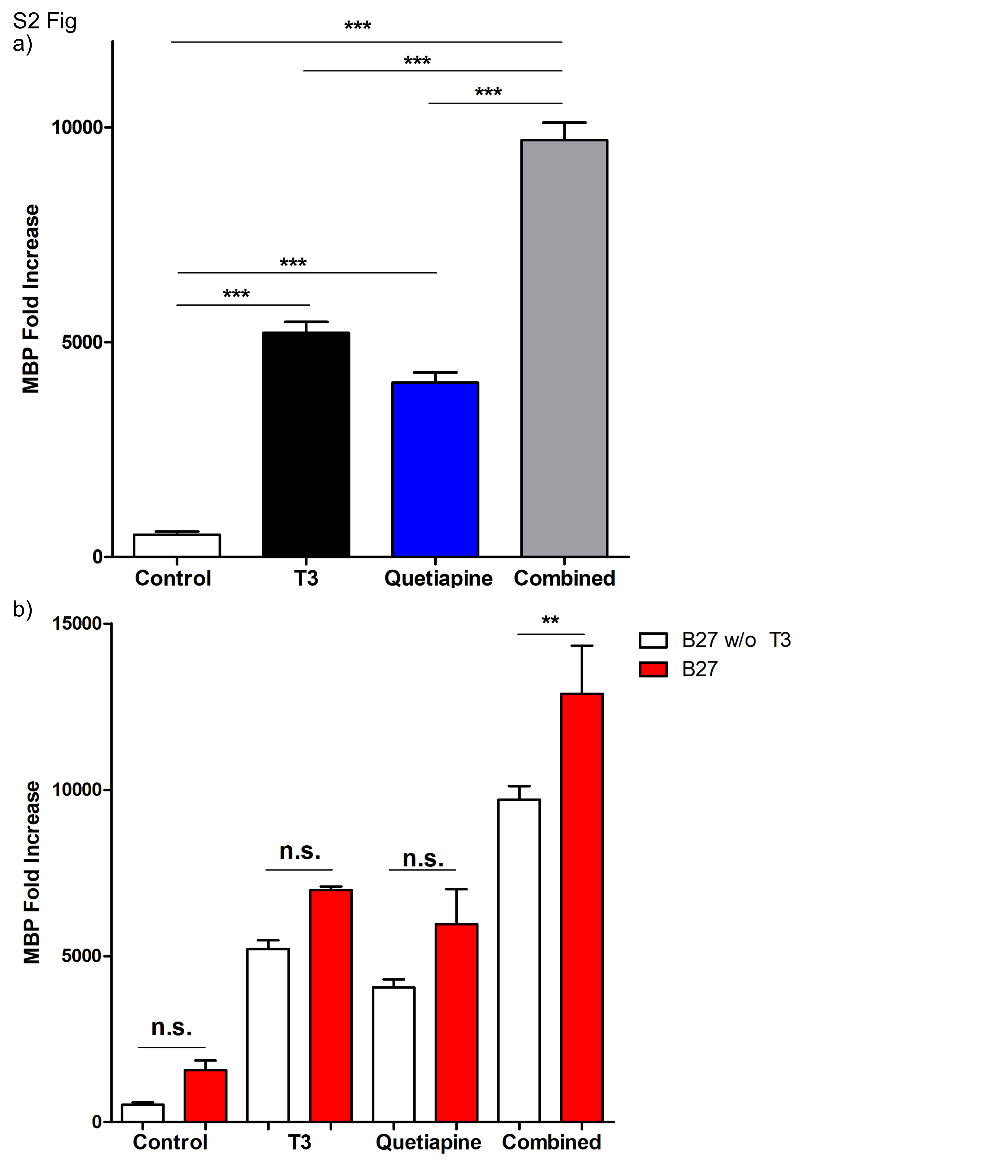

Supplement: S2 Fig — OPCs isolated from 4–7 day old rats were cultured for 96 hours with PDGF 20ng/ml in OPC media made with all of the components of B27 except T3. (a) After 96 hours, either T3 45nM (Black), Quetiapine 1μM (Blue), or both (Grey) was added to the media for an additional 96 hrs. OPC media with 0.1%DMSO (vehicle) was used as control (White). MBP expression was measured by qPCR. A pre-treatment day 0 sample was used to normalize gene results. Error bars represent standard error of the mean from 2 independent isolations and experiments. One-way ANOVA analysis with Tukey’s multiple comparison analysis was run (*** p< 0.0001). (b) Comparison between OPCs treated in media with either B27 (Red) supplement (as in Fig 1a) or with all of the components of B27 except T3 (White) (as in S2a Fig). MBP expression was measured by qPCR. A pre-treatment day 0 sample was used to normalize gene results. Significance was determined using a One-way ANOVA analysis with Tukey’s multiple comparison analysis (n.s. non significant, ** p< 0.001). (TIF) [file pone.0221747.s002.TIF]

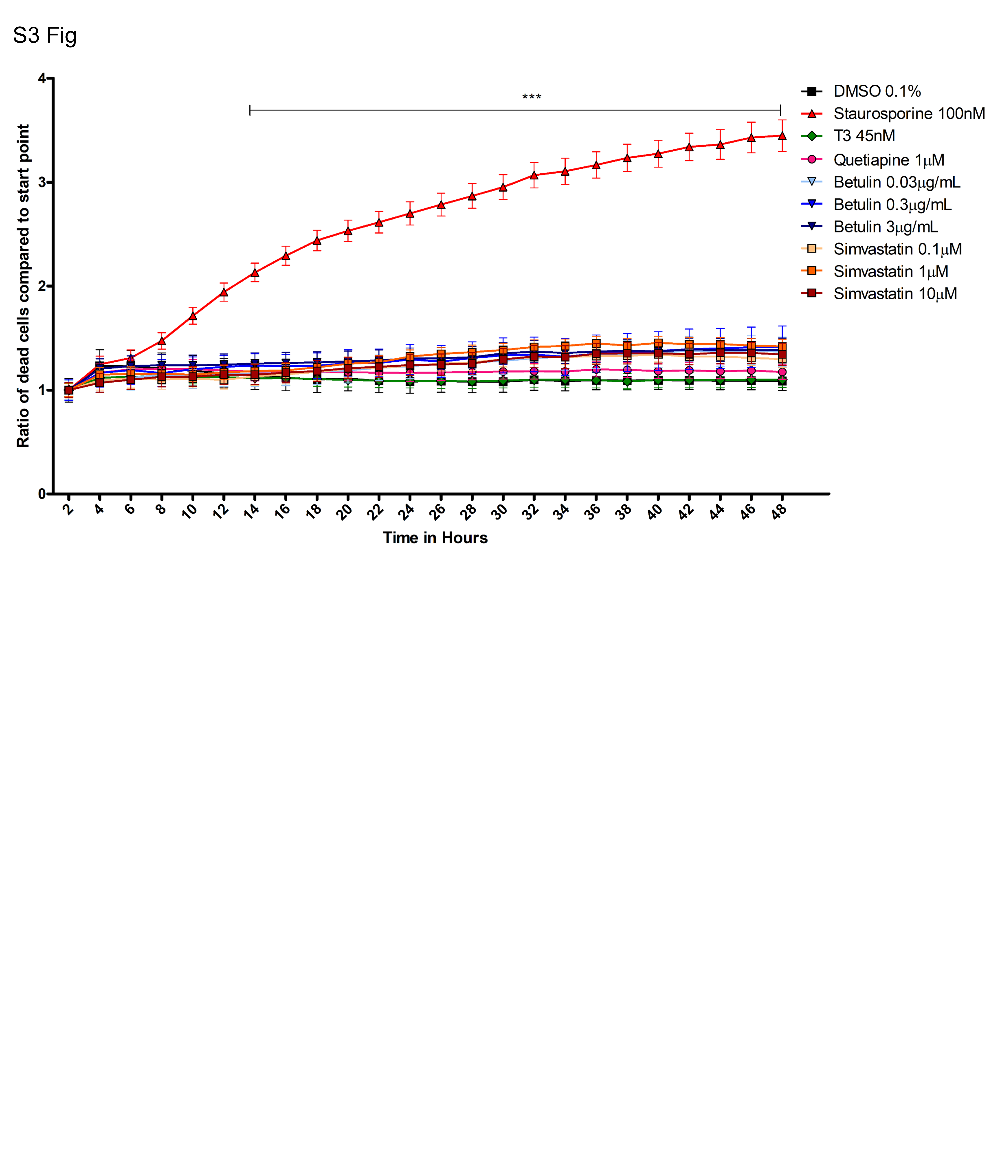

Supplement: S3 Fig — Cell toxicity assays of each compound were performed on cultured OPCs in the presence of IncuCyte Cytotox Green Reagent. (a) Ratio of number of dead cells at each time point by number of dead cells at the beginning of the experiment were calculated. Error bars represent standard error of the mean. 2-way ANOVA analysis with Tukey’s post tests were run (*** p < 0.0001 for stausporin compared to vehicle DMSO 0.1%). (TIF) [file pone.0221747.s003.TIF]

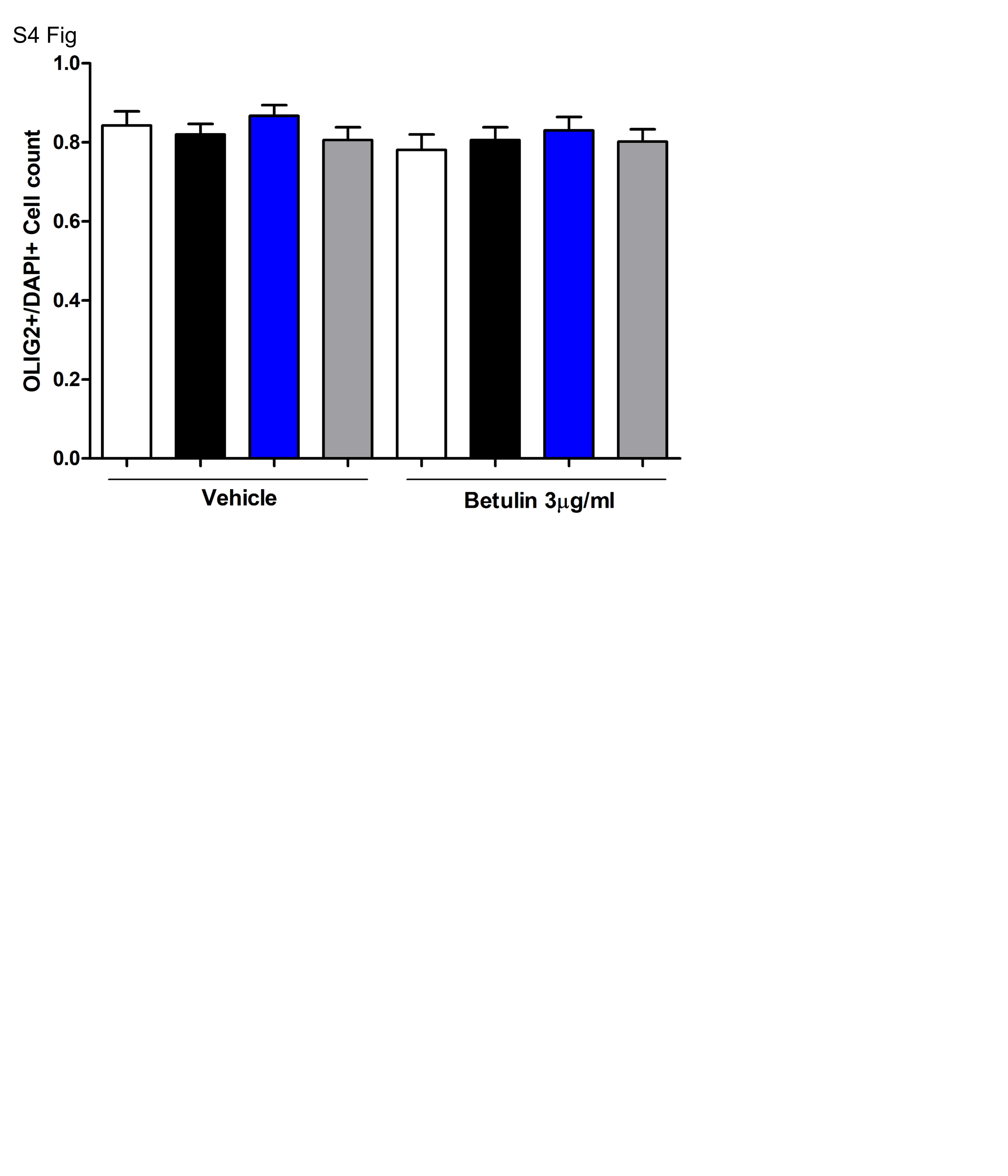

Supplement: S4 Fig — OLIG2 and DAPI positive cells were enumerated and the ratio is shown for each condition, T3 45nM (Black), Quetiapine 1μM (Blue), or both (Grey) for 96 hrs. OPC media with 0.1%DMSO (vehicle) was used as control (White) in the presence or absence of betulin 3 μg/ml. Error bars represent standard error of the mean. A one-way ANOVA analysis showed no significant difference. (TIF) [file pone.0221747.s004.TIF]
